# Morbid Anatomy of Milksickness

**Published:** 1841-09

**Authors:** 


### MORBID ANATOMY OF MILKSICKNESS.

We have received from Dr. J. V. Wagman of New Castle, Ind., an account of the dissection of a man who died of milksickness. The history of the case is not given, but the Doctor is familiar with the disease, and regarded this case as marked with the pathognomic symptoms. The patient, 30 years old, of regular habits and sanguine temperament, had a vigorous constitution, and enjoyed sound health up to the time of the attack. During the progress of the disease, he took no medicine, till a few hours before his death, when all that he swallowed was immediately ejected; so that whatever morbid appearances existed, might, as the Doctor remarks, be legitimately referred to the disease of which he died. The dissection was made fifteen hours after death.

The body was not much emaciated. The skin had a dusky yellowish hue.

The brain and its membranes exhibited nothing remarkable, except perhaps more than the usual quantity of serum in the ventricles.

The stomach presented a number of patches of light brown and scarlet colors mixed. In some places the mucous membrane was thickened and soft. The pyloric orifice was of a scarlet hue. The mucous membrane of the duodenum presented the same kind of patches with that of the stomach; and some parts were dry. The bowel itself, as well as the lower part of the stomach, was much contracted. The other small intestines were pale; the mucous membrane was softened, many portions of it were dry; the glands of Peyer & Brunner were swollen and soft, and some of them appeared to be ulcerated. The cœcum way dry. The colon contained hardened fæces, on which it contracted closely; was drier than other portion of the tube; its color was a dark brown, with rose colored patches. The liver was of a dark color and seemed unusually friable under pressure by the fingers; the gall-bladder was much distended with a black pitchy bile. The pancreas was of a rose color and appeared rather soft. The spleen was much enlarged, of a deep brown color, and very soft. The peritoneum had reddish spots, and there was some increased effusion into its cavity. The kidneys, bladder, heart and lungs were sound.

It is proper, to say, that Dr. W. modestly intimates, that his experience in *post mortem* inspections has not been sufficiently extensive, to give him confidence in his own observations. From our knowledge of him, we have no doubt that he has aimed to make an accurate report of the case. We hope his brethren, in regions where the sick-stomach is endemic, will, as often as possible, follow his example, and like him favor us with the results of their examinations.

